# Feasibility of apalutamide combined with androgen deprivation therapy and short-course low-dose prednisone in treating metastatic hormone-sensitive prostate cancer: a pilot randomized controlled trial

**DOI:** 10.3389/fonc.2023.1110807

**Published:** 2023-11-06

**Authors:** Dingyuan Yang, Wenqiang Chen, Fei Lai, Mingxing Qiu, Jun Li

**Affiliations:** ^1^ Urinary Surgery, Chengdu Second People’s Hospital, Chengdu, China; ^2^ Urinary Surgery, Sichuan Academy of Medical Sciences, Sichuan People’s Hospital, Chengdu, China

**Keywords:** apalutamide, metastatic hormone-sensitive prostate cancer, prednisone, randomized controlled trial, rash

## Abstract

**Introduction:**

The role of prednisone in the prevention of androgen receptor antagonist-related rash and treatment for metastatic hormone-sensitive prostate cancer (mHSPC) is unclear. This pilot trial (ChiCTR2200060388) aimed to investigate the feasibility of apalutamide combined with androgen deprivation therapy (ADT) and short-course low-dose prednisone in the treatment of mHSPC.

**Methods:**

All patients received apalutamide and ADT and were randomly divided into two groups based on the administration of oral prednisone or not (control group). The primary endpoint was the incidence of rash. The secondary endpoint included the proportions of patients with a decline in PSA ≥50% from baseline, PSA ≥90% from baseline, and decreased to PSA ≤0.2 ng/mL.

**Results:**

Between June 2021 and March 2022, a total of 83 patients were enrolled (41 in the prednisone group and 42 in the control group). During the 6-month follow-up, the incidence of rash was significantly lower in the prednisone group compared with the control group (17.1% vs. 38.1%, *P*=0.049). There were no significant differences in the incidence of other adverse events, the number of patients who required dose adjustment (reduction, interruption, or discontinuation) of apalutamide due to rash, the number of patients with prostate-specific antigen (PSA) decreased by ≥50%, the number of patients with PSA decrease ≥90%, and the number of patients with PSA ≤0.2 ng/mL between the two groups. All patients with diabetes had stable glycemic control with no glucose-related adverse events.

**Discussion:**

In patients with mHSPC, the addition of short-course low-dose prednisolone to apalutamide plus ADT can reduce the incidence of rash without risk of other adverse events.

## 1 Introduction

Prostate cancer is the most prevalent malignant tumor in the male genitourinary system worldwide and has become an important public health problem that requires global attention (1). With the aging and lifestyle changes in the Chinese population, the incidence of prostate cancer is rising, and 70% of patients were diagnosed with locally advanced disease or extensive metastasis (2). There are many treatment methods for prostate cancer, such as surgery, endocrine therapy, radiotherapy, and chemotherapy (3). Among them, androgen deprivation therapy (ADT) is still the mainstay treatment. For metastatic prostate cancer, 80%-90% of patients have a favorable response to ADT, defined as metastatic hormone-sensitive prostate cancer (mHSPC) (4). However, as the disease progresses further, almost all mHSPC will transition to metastatic castration-resistant prostate cancer (mCRPC), leading to poor prognosis and high mortality. Therefore, how to effectively treat mHSPC to prevent or delay the progression to mCRPC and then to improve the prognosis and reduce the mortality is a key issue in this field.

The NCCN guidelines and EAU guidelines recommend 1) ADT combined with abiraterone, apalutamide, or enzalutamide, 2) ADT combined with docetaxel with abiraterone or darolutamide, 3) ADT with external beam radiotherapy, or 4) ADT alone as first-line treatment for mHSPC (5, 6). However, chemotherapy initiation, such as docetaxel, is often influenced by the patient’s age and the extent of the disease, and the patient’s willingness. The use of abiraterone requires co-administrated with prednisone to prevent the occurrence of corticotropin elevation and other drug-related adverse events.

The most common mechanism of antiandrogens resistance involves overexpression, amplification, and/or mutation of AR, expression of AR splice variants, increased production of intracellular androgens, and changes in the activity or expression of AR co-activators and co-repressors (7, 8). Apalutamide is a new-generation androgen receptor (AR) antagonist that binds directly to AR and inhibits downstream AR-mediated transcription of prostate cancer-related genes (4). Currently, apalutamide combined with ADT has become the first-line treatment option for mHSPC (9, 10). Regarding the safety of apalutamide, a certain proportion of patients with mHSPC in the TITAN study had dose reductions (7.1%), interruptions (19.8%), or discontinuations (8.0%) of treatment due to adverse events (AEs), with the most common being rash (11). The incidence of any-grade rash in the apalutamide group was higher than in the placebo group (27.1% versus 8.5%) (11). The incidence of grade 3 or above rash was 6.3% in the apalutamide group, and the median time to the occurrence of rash was 81 days (11). In addition, drug-related rashes are graded based on the body surface area (BSA) rather than severity. Thus, grade 3 rash should cover over 30% of BSA. The TITAN study showed effective relief of rash after the use of glucocorticoids (11). In addition, the rate of maculopapular rash was lower in the abiraterone plus prednisone group than in the abiraterone group (9% vs. 14%) (12), indicating that prednisone is effective for drug-related rash. However, there are no prior studies on the topic of preventing apalutamide-related rashes. In addition to anti-allergic effects, glucocorticoids have also been found to have anti-tumor effects (13). However, whether prednisone combined with apalutamide and ADT can improve the anti-tumor efficacy of mHSPC compared with apalutamide and ADT has not been further investigated.

In this pilot trial, we aimed to preliminarily investigate whether prednisone can prevent drug-related rash in patients with mHSPC treated with apalutamide and ADT and to evaluate the safety and feasibility of prednisone combined with apalutamide and ADT in the treatment of mHSPC.

## 2 Methods

### 2.1 Study design and participants

This multicenter, open-label, randomized controlled pilot trial (ChiCTR2200060388) was conducted at Sichuan Province People’s Hospital and Chengdu Second People’s Hospital between June 2021 and March 2022. The inclusion criteria were: 1) metastatic prostate cancer confirmed by pathological examination; 2) computed tomography (CT), bone scan, or magnetic resonance imaging confirmed distant metastasis; 3) hormone-sensitive disease, defined as radiographic or prostate-specific antigen (PSA) response to ADT and antiandrogen therapy; 4) life expectancy >3 months; and 5) adequate blood function: absolute neutrophil count ≥2×10^9^/L, platelet count ≥100×10^9^/L, hemoglobin ≥9 g/dL, total bilirubin ≤ upper limit of normal (ULN); 6) adequate liver function: aspartate aminotransferase and alanine aminotransferase ≤2.5×ULN, alkaline phosphatase ≤5×ULN; 7) adequate renal function: serum creatinine ≤ULN or creatinine clearance ≥60 mL/min. Prior radical surgery or radiotherapy for prostate cancer was allowed, and radiotherapy should be completed prior to enrollment. Prior ADT for up to 6 months was allowed. The exclusion criteria were: 1) neuroendocrine carcinoma, prostate small cell carcinoma, or ductal carcinoma; 2) contraindication to prednisone; 3) any comorbid chronic disease requiring treatment with doses of prednisone greater than 5 mg once daily; 4) history of hypersensitivity or intolerance to any drugs used in the study; 5) patients who had fertility but were unwilling to use effective contraception; 6) any uncontrolled systemic disease, including active infection, active autoimmune disease and uncontrolled cardiovascular disease (such as hypertension, diabetes, myocardial infarction, and cerebral infarction); 7) other malignancies within the past 5 years; poor compliance and inability to cooperate with treatment and follow-up; or 8) participation in other clinical trials within the last 3 months.

The study was approved by the ethics committee of each participating center. Written informed consent was obtained from each patient.

### 2.2 Treatment

Patients were randomly divided into the prednisone group and control group using a random number table. The treatment cycle was 28 days, and treatment continued until progression. The ADT regimen remained unchanged during treatment. The dosage of drugs was adjusted based on adverse reactions, and the drugs could be suspended for no more than four weeks.

Both groups received oral apalutamide 240 mg once daily and an ADT regimen that adopted gonadotropin-releasing hormone analog (GnRHa), including GnRHa agonists and antagonists. The prednisone group additionally received oral prednisone 5 mg once daily for 3 months. The dose of apalutamide could be reduced to 120 mg/d. Apalutamide treatment could be suspended for 2 weeks, followed by restart at a dose of 120 mg/d.

The treatment protocol for rashes depended upon the grade. Grade 1-2: the dose of apalutamide was reduced, or apalutamide was stopped, oral antihistamines + topical or oral steroids were given, and, if necessary, a dermatology consultation was requested to assist in the management. Grade 3-4: apalutamide was suspended or stopped, oral antihistamines + topical and oral steroids were given, and a dermatologic consultation was requested to assist with treatment if necessary. The effect of the rash treatment was reevaluated after 1 week of treatment, and the treatment plan was decided according to the severity of the rash.

### 2.3 Data collection

Age, Gleason score, Eastern Cooperative Oncology Group (ECOG) score, medical history, baseline PSA, and extent of metastases (high volume [defined as the presence of visceral metastases or ≥4 bone lesions with ≥1 beyond the vertebral bodies and pelvis or presence of visceral metastases] vs. low volume) according to the CHAARTED criteria (14) were collected at enrollment.

The diabetic patients enrolled in this study were monitored during treatment for fasting and 3-h postprandial glucose. The serum PSA levels were measured every 28 days. Imaging examinations such as bone scans and whole-abdominal enhanced CT were performed every 3 months. The data cut-off date was March 2022.

### 2.4 Endpoints

The primary endpoint was the incidence of rash. Secondary endpoints included the incidence of rash-induced dose reduction, the incidence of rash-induced drug interruption, the incidence of rash-induced drug discontinuation, the proportion of patients with a decline in PSA ≥50% from baseline, the proportion of patients with a decline in PSA ≥90% from baseline, the proportion of patients decreased to PSA ≤0.2 ng/mL, and the incidence of treatment-emergent AEs.

### 2.5 Statistical analysis

SPSS 26.0 statistical software (IBM Corp., Armonk, NY, USA) was used for statistical analysis. Categorical variables were presented as frequencies (%), and the chi-square test was used for comparison between groups. PSA responses (decline ≥50%, decline ≥90%, or decrease to ≤0.2 ng/mL) were analyzed using a waterfall plot, and the chi-square test was used for comparison between groups. *P*<0.05 was considered statistically significant.

## 3 Results

### 3.1 Baseline characteristics

A total of 83 patients were randomly assigned to the prednisone group (n=41) and the control group (n=42). Demographic and clinical characteristics at baseline were comparable (*P* > 0.05). There are 9.8% and 4.8% of patients aged < 70 years in the prednisone group and control group, respectively, whereas 90.2% and 95.2% of patients aged ≥ 70 years in the two groups. 36.6% of patients (n=15) had a Gleason score of <8 and 63.4% (n=26) had a Gleason score of ≥8 in the study arm, while 47.6% of patients (n=20) had a Gleason score of <8 and 52.4% (n=22) had Gleason score ≥8 in the control arm. There were six (15.6%) and nine (21.4%) patients with diabetes mellitus in the prednisone and control groups, respectively (*P*=0.570). The detailed information is reported in **Table 1**.

**Table 1:** Baseline characteristics.

Characteristics, n (%)	Apalutamide+ADT+ prednisone (n=41)	Apalutamide+ADT (n=42)	*P*
Age (years)			0.433
<70	4 (9.8)	2 (4.8)	
≥70	37 (90.2)	40 (95.2)	
Diabetes mellitus	6 (15.6)	9 (21.4)	0.570
Gleason score			0.309
<8	15 (36.6)	20 (47.6)	
≥8	26 (63.4)	22 (52.4)	
ECOG score			0.202
0-2	37 (90.2)	41 (97.6)	
>2	4 (9.8)	1 (2.4)	
Baseline PSA (ng/mL)			0.750
<100	19 (46.3)	18 (42.9)	
≥100	22 (53.7)	24 (57.1)	
Extent of metastases			0.740
High volume	27 (65.9)	26 (61.9)	
Low volume	14 (34.1)	16 (38.1)	

ECOG, Eastern Cooperative Oncology Group; PSA, prostate-specific antigen.

### 3.2 Safety

The common AEs in the prednisone and control groups were rash, fatigue, hot flush, hypertension, gastrointestinal events (diarrhea or nausea), and cardiovascular events. There were seven patients (17.1%) with rash in the prednisone group and 16 (38.1%) with rash in the control group, with a statistically significant difference (*P*=0.049). The median time of rash occurrence was 73 days.

In the prednisone group, three patients had grade 1-2 rash: two received steroids 10 mg tid for 1 week and achieved improvement; one received steroids 10 mg tid for 1 week, and rashes showed no improvement, steroids were increased to 15 mg for 1 week without improvement, and then apalutamide was stopped. In the control group, five patients had grade 1-2 rash: three received steroids 10 mg tid for 1 week and achieved improvement; two received steroids 10 mg tid for 1 week without improvement, steroids were increased to 15 mg for 1 week, and then the rashes improved. In the prednisone group, one patient had a grade ≥3 rash; he received steroids 15 mg tid for 2 weeks, and the rash improved. In the control group, three patients had grade ≥3 rash; they were treated with steroids 15 mg tid for 2 weeks; one improved, one showed no improvement, and one was unknown.

The difference in the incidence of fatigue, hot flush, hypertension, gastrointestinal events, or cardiovascular events between the two groups was not statistically significant (*P*>0.05). A comparison of common AEs in the two groups is shown in **Table 2**.

**Table 2:** Common adverse events.

Events, n (%)	Any Grade			Grade 3/4		
	Apalutamide+ADT+ Prednisone (n=41)	Apalutamide+ADT	*P*	Apalutamide+ADT+ Prednisone (n=41)	Apalutamide+ADT	*P*
		(n=42)			(n=42)	
Rash	7 (17.1)	16 (38.1)	0.049	1 (2.4)	3 (7.1)	0.616
Fatigue	8 (19.5)	10 (23.8)	0.791	2 (4.9)	2 (4.8)	1.000
Hot flush	7 (17.1)	6 (14.3)	0.771	0	0	/
Hypertension	5 (7.3)	4 (9.5)	0.738	2 (4.9)	1 (2.4)	1.000
Gastrointestinal events	5 (12.2)	6 (14.3)	1.000	0	0	/
Cardiovascular events	2 (4.9)	1 (2.4)	0.616	0	0	/

The difference in dose adjustment of apalutamide after the onset of rash between the two groups was not statistically significant (*P*>0.05), as shown in **Table 3**.

**Table 3:** Dose adjustment of apalutamide due to rash.

Dose adjustment, n (%)	Apalutamide+ADT+ Prednisone (n=41)	Apalutamide+ADT (n=42)
Dose reduction	2 (4.9)	2 (4.8)
Interruption	1 (2.5)	3 (7.1)
Discontinuation	1 (2.5)	1 (2.4)

All patients with diabetes had stable glycemic control with no glucose-related adverse events.

### 3.3 PSA response

After 6 months of treatment, the number of patients with a PSA decrease of ≥50% was 39 (95.1%) in the prednisone group and 39 (92.9%) in the control group; the number of patients with a PSA decrease of ≥90% was 35 (85.4%) in the prednisone group and 33 (78.6%) in the control group; the number of patients with PSA decreased to ≤0.2 ng/mL was 33 (80.5%) in the prednisone group and 32 (76.2%) in the control group. None of the differences in PSA response was statistically significant between the two groups (*P*>0.05).

**Figure 1:**
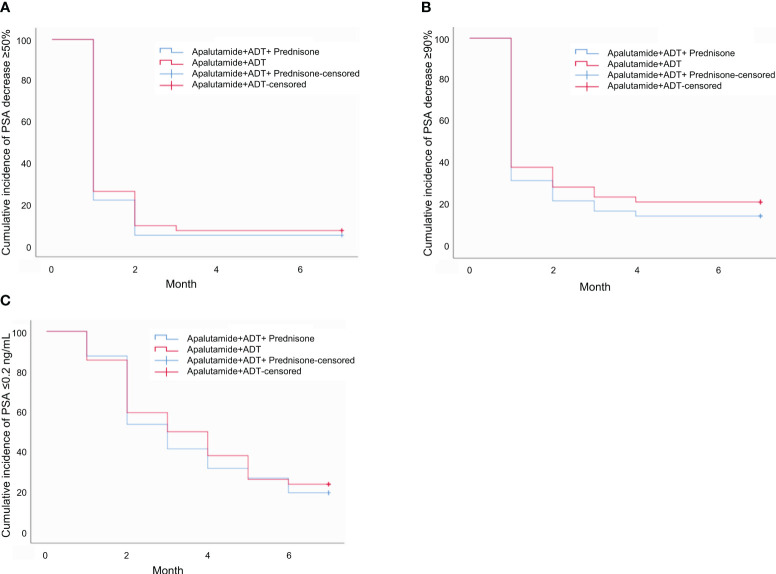
**(A)** Time to PSA decrease ≥50%. **(B)** Time to PSA decrease ≥90%. **(C)** Time to PSA ≤0.2 ng/mL.

None of the differences in the median time to PSA response was statistically significant between the prednisone group and the control group (*P*>0.05), as shown in **Table 4** and **Figures 1A-C**.

**Table 4:** PSA response after 6 months of treatment.

Endpoints	Apalutamide+ADT+ Prednisone (n=41)	Apalutamide+ADT (n=42)	*P*
PSA decrease ≥50%, n (%)	38 (92.7)	39 (92.9)	1.000
Median time to onset, months (95%CI)	1 (1,1)	1 (1,1)	0.794
PSA decrease ≥90%	36 (87.8)	32 (76.2)	0.169
Median time to onset, months (95%CI)	1 (2,1)	1 (3,1)	0.243
PSA ≤0.2 ng/mL	24 (58.5)	21 (50.0)	0.435
Median time to onset, months (95%CI)	3 (-,2)	3 (-,2)	0.654

PSA, prostate-specific antigen; CI, confidence interval.

**Figure 2:**
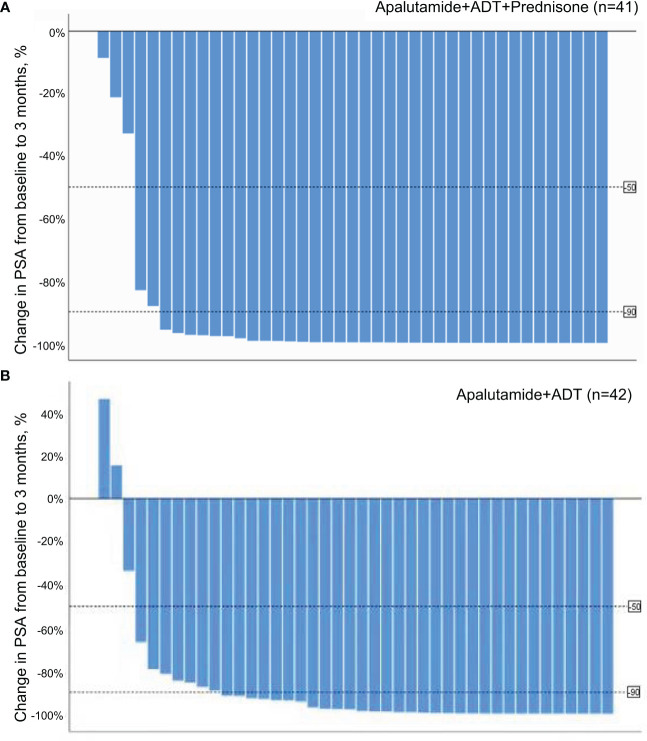
**(A)** Waterfall plot of PSA response at 3 months in the treatment group. **(B)** Waterfall plot of PSA response at 3 months in the control group.

Waterfall plots in **Figure 2A** show PSA decrease for all patients in the treatment group, whereas two patients had a PSA increase in the control group.

## 4 Discussion

The international, randomized phase III TITAN trial confirmed that apalutamide plus ADT could significantly prolong overall survival (OS) and radiographic progression-free survival in patients with mHSPC compared with placebo plus ADT (11), thus was recommended by the NCCN guidelines as a first-line regimen for mHSPC (5). In TITAN, the most common AE with apalutamide plus ADT was rash (27.1%), and 5.3%, 8.4%, and 2.3% of patients had rash leading to dose reductions, interruptions, and discontinuations (11), respectively. In the Eastern Asian subpopulation of TITAN, the rash rate was higher (37.3%) (15). Rash not only causes direct damage to patients’ health but also affects treatment compliance to apalutamide, thereby affecting the anti-tumor effect and patient prognosis. Therefore, the prevention of rash has great significance in clinical practice. This multicenter, open-label, randomized controlled trial investigated the addition of short-course low-dose prednisone to apalutamide plus ADT in patients with mHSPC. The results showed that prednisone could prevent rash during treatment with apalutamide and ADT, with decreased incidence of rash compared with the control group (17.1% vs. 38.1%). In addition, the addition of prednisone did not affect the efficacy of apalutamide plus ADT, with similar PSA response between the two groups.

Prednisone can exert anti-inflammatory, anti-allergic, and immunosuppressive effects by binding to the cytoplasmic glucocorticoid receptor and can be used to treat various allergic diseases, bacterial infections, eczema, systemic lupus erythematosus, exfoliative dermatitis, etc. (16). Previous studies demonstrated that rash resulted from abiraterone (12), chemotherapy (17), endocrine therapy (18), and anti-human immunodeficiency virus therapy (19) could be prevented by the use of glucocorticoids. It is suggested that apalutamide may induce rash through immune mechanisms such as delayed allergic reactions. The experimental results of drug allergy mouse models confirmed that apalutamide 2-cyanopyridine reacts with cysteine in proteins to form semi-antigens and triggers an immune response (20). Therefore, prednisone may prevent or treat apalutamide-related rash by suppressing the immune response mechanism. The TITAN study showed that rash could be effectively relieved if patients used glucocorticoids at the time of rash onset (11). In our study, the incidence of rash was significantly reduced with the addition of prednisone to apalutamide plus ADT, and grade 3-4 rash only accounted for 2.5% in the prednisone group. This suggests that prednisone can prevent the occurrence of rash and reduce the risk of severe rash during the treatment of apalutamide plus ADT in patients with mHSPC. In addition, our results also showed that the addition of prednisone did not increase the risk of other AEs.

In addition to the anti-inflammatory effects of glucocorticoids, their anti-tumor effects, such as on malignant hematological tumors, breast cancer, and prostate cancer, are gradually being revealed (13). Previous studies have found that PSA level is a powerful tool for predicting disease progression in prostate cancer, helping provide the first signal for regimen adjustment and guiding optimal treatment decisions for prostate cancer in advance. It is not enough to focus on the occurrence of PSA decline alone; we need to focus on meaningful PSA decline. The lower the PSA level and the faster it reaches the valley value, the longer OS it predicts (21, 22). How to rapidly, deeply, and durably reduce PSA levels to delay disease progression, prolong survival, and better achieve the treatment goal of patients with mHSPC has become a research hotspot. In our study, PSA response was reflected by three definitions (PSA decrease ≥50%, PSA decrease ≥90%, and PSA ≤0.2 ng/mL). The difference in PSA response was not significantly different between the two groups, regardless of the definition. Given that the proportion of patients with PSA response was numerically higher in the prednisone group compared with the control group (85.4% vs. 78.6%) when PSA response was defined as PSA decrease ≥90%, the relatively small sample size might be a possible reason that leads to an underpowered statistic. Moreover, since there were no previous studies on prednisone combined with apalutamide, our study used prednisone 5 mg once daily for 3 months to avoid the side effects associated with long-course high-dose administration. In addition, some *in vitro* studies have observed that glucocorticoids, in combination with various cytotoxic anticancer drugs and radiotherapy, may induce treatment resistance when used in cultured cells or xenograft mouse models. This phenomenon has been found in many epithelial cancer types, including prostate, kidney, testicular, and bladder cancers (23), raising concerns when glucocorticoids are used in combination treatment regimens. In our study, although we did not find an improvement in PSA response in the prednisone group, the addition of prednisone at least did not affect the efficacy of apalutamide plus ADT. The optimal administration regimens of prednisone and its anti-tumor effect need to be further investigated, and large-scale trials are warranted.

The analysis of the Eastern Asian subpopulation from the TITAN study showed that the median percentage of PSA decline at 12 weeks from baseline was 96.1%, and the median maximum percentage of PSA decline at any timepoint from baseline was 99.0% in the apalutamide group (15). In our study, a large proportion of patients achieved PSA response (≥50% or ≥90%) in both groups, with a median time of 1 month. Approximately 80% of patients in both groups could achieve PSA ≤0.2 ng/mL after 6 months of treatment. Similar to previous reports (15), our study also showed a rapid and deep PSA response with apalutamide plus ADT in patients with mHSPC.

Oishi et al. (24) concluded that a dose reduction of apalutamide was not associated with differences in the incidence of skin-related AEs between the standard- and reduced-dose groups. Still, based on the subgroup analysis, they also concluded that apalutamide dose reduction in patients with small body sizes could decrease the occurrence of skin-related AEs without sacrificing the oncological outcomes. These results could have important implications for Asian patients, who are smaller than other ethnic groups (25, 26). The study by Oishi et al. (24) was not published when the present trial was designed, but it should be considered in the design of future trials. Still, the previous study was relatively small, and it should also be carefully examined whether a reduced dose can be given to Asian patients without efficacy issues.

There are some limitations in this study. The sample size was relatively small and not pre-calculated because this study was a pilot trial, and the follow-up duration was short. Nevertheless, it provides valuable data for the design of future large-scale trials. Further large-scale randomized controlled trials are warranted to validate our findings and confirm the anti-tumor effect of prednisone when combined with apalutamide and ADT.

In conclusion, prednisone combined with apalutamide and ADT is feasible in reducing the incidence of rash and possibly reducing the severity of rash in patients with mHSPC without increasing the incidence of other AEs. The anti-tumor effect of prednisone needs further exploration when used in combination with apalutamide and ADT.

## Data availability statement

The original contributions presented in the study are included in the article/supplementary material. Further inquiries can be directed to the corresponding author.

## Ethics statement

The studies involving human participants were reviewed and approved by the ethics committee of each participating center. The patients/participants provided their written informed consent to participate in this study.

## Author contributions

DY conceived and coordinated the study, designed, performed and analyzed the experiments, wrote the paper. WC, FL and MQ carried out the data collection, data analysis, and revised the paper. JL conceived and coordinated the study, carried out the data analysis, and revised the paper. All authors contributed to the article and approved the submitted version.
